# Missed Opportunities for Screening and Management of Dysglycemia among Patients Presenting with Acute Myocardial Infarction in North India: The Prospective NORIN STEMI Registry

**DOI:** 10.5334/gh.1140

**Published:** 2022-08-12

**Authors:** John W. Ostrominski, Muthiah Vaduganathan, Meennahalli Palleda Girish, Puneet Gupta, Michael J. Hendrickson, Arman Qamar, Sameer Arora, Ambarish Pandey, Ankit Bansal, Vishal Batra, Bhawna Mahajan, Saibal Mukhopadhyay, Jamal Yusuf, Sanjay Tyagi, Deepak L. Bhatt, Mohit D. Gupta, NORIN STEMI Investigators

**Affiliations:** 1 Brigham and Women’s Hospital Heart and Vascular Center, Harvard Medical School, Boston, MA, USA; 2 Department of Cardiology, Gobind Ballabh Pant Institute of Postgraduate Medical Education and Research, New Delhi, India; 3 Department of Cardiology, Janakpuri Superspeciality Hospital, New Delhi, India; 4 Division of Cardiology, University of North Carolina School of Medicine, Chapel Hill, NC, USA; 5 NorthShore Cardiovascular Institute, NorthShore University Health System, University of Chicago Pritzker School of Medicine, Evanston, Illinois, USA; 6 Division of Cardiology, Department of Internal Medicine, University of Texas Southwestern Medical Center, Dallas, USA; 7 Department of Biochemistry, Gobind Ballabh Pant Institute of Postgraduate Medical Education and Research, New Delhi, India

**Keywords:** diabetes, cardiometabolic, screening, prevention, low- and middle-income countries, myocardial infarction

## Abstract

**Background:**

Dysglycemia is a major and increasingly prevalent cardiometabolic risk factor worldwide, but is often undiagnosed even in high-risk patients. We evaluated the impact of protocolized screening for dysglycemia on the prevalence of prediabetes and diabetes among patients presenting with ST-segment elevation myocardial infarction (STEMI) in North India.

**Methods:**

We conducted a prospective NORIN STEMI registry-based study of patients presenting with STEMI to two government-funded tertiary care medical centers in New Delhi, India, from January to November 2019. Hemoglobin A1c (HbA1c) was collected at presentation as part of the study protocol, irrespective of baseline glycemic status.

**Results:**

Among 3,523 participants (median age 55 years), 855 (24%) had known diabetes. In this group, baseline treatment with statins, sodium-glucose cotransporter 2 inhibitors, or glucagon-like peptide-1 receptor agonists was observed in 14%, <1%, and 1% of patients, respectively. For patients without known diabetes, protocolized inpatient screening identified 737 (28%) to have prediabetes (HbA1c 5.7–6.4%) and 339 (13%) to have newly detected diabetes (HbA1c ≥ 6.5%). Patients with prediabetes (49%), newly detected diabetes (53%), and established diabetes (48%) experienced higher rates of post-MI LV dysfunction as compared to euglycemic patients (42%). In-hospital mortality (5.6% for prediabetes, 5.1% for newly detected diabetes, 10.3% for established diabetes, 4.3% for euglycemia) and 30-day mortality (8.1%, 7.6%, 14.4%, 6.6%) were higher in patients with dysglycemia. Compared with euglycemia, prediabetes (adjusted odds ratio (aOR) 1.44 [1.12–1.85]), newly detected diabetes (aOR 1.57 [1.13–2.18]), and established diabetes (aOR 1.51 [1.19–1.94]) were independently associated with higher odds of composite 30-day all-cause mortality or readmission.

**Conclusions:**

Among patients presenting with STEMI in North India, protocolized HbA1c screening doubled the proportion of patients with known dysglycemia. Dysglycemia was associated with worse clinical outcomes at 30 days, and use of established pharmacotherapeutic risk-reduction strategies among patients with known diabetes was rare, highlighting missed opportunities for screening and management of dysglycemia among high-risk patients in North India.

## Introduction

Dysglycemia, including prediabetes and diabetes, is a major and increasingly prevalent risk factor for cardiovascular disease and mortality worldwide [1,2]. As of 2019, the estimated global prevalence of diabetes in adults aged 20–79 years was 463 million and is projected to increase by more than 50% to over 700 million by 2045 [2]. An estimated 80% of patients with diabetes reside in low- and middle-income countries (LMICs), where the prevalence of prediabetes is also rising and contributes independently to excess cardiovascular risk and all-cause mortality [2,3,4].

Despite the robust contribution of diabetes to cardio-renal-metabolic risk, it is often undiagnosed, even among high-risk patients with established cardiovascular disease [5]. The rate of undiagnosed diabetes is especially high in LMICs; the estimated proportion of undiagnosed diabetes in India is among the highest globally, amounting to 43 million persons [2]. As cardiovascular risk reduction strategies have been established for diabetes, namely sodium-glucose cotransporter 2 inhibitors (SGLT2i) and glucagon-like peptide-1 receptor agonists (GLP-1RA) [6], undiagnosed diabetes constitutes an important missed opportunity for primary and secondary prevention of diabetes-related complications. However, little is known about the uptake of these therapies in LMICs.

Among patients presenting with STEMI in North India in the prospective North Indian ST-Segment Myocardial Infarction (NORIN STEMI) registry, we evaluate the impact of protocolized dysglycemia screening on the prevalence of dysglycemia and its association with clinical outcomes. We also examine the frequency with which patients presenting with STEMI and known diabetes are treated with SGLT2i and GLP-1RA at baseline.

## Methods

### NORIN STEMI registry and participants

The aims and design of the prospective NORIN STEMI registry have been previously described [7]. Briefly, patients presenting with STEMI to two government-funded tertiary care medical centers in New Delhi, India were enrolled. Patients aged ≤18 years or presenting more than 21 days following symptom onset were excluded. STEMI was defined according to the Fourth Universal Definition of Myocardial Infarction [8].

### Protocolized dysglycemia screening

Glycated hemoglobin (HbA1c) was collected as part of the study protocol, irrespective of baseline glycemic status, and estimated by D-10 ion-exchange high-performance liquid chromatography (Bio-Rad Laboratories, California, USA). Diabetes status was established on admission based on patient interview and chart review. Baseline medications were reviewed for all participants. In this analysis, patients were stratified into four groups: euglycemia, prediabetes, newly detected diabetes, and established diabetes. Euglycemia was defined as screening HbA1c <5.7%; prediabetes and diabetes were defined as screening HbA1c 5.7–6.4% and ≥6.5%, respectively. Established diabetes was defined as a history of diabetes per either patient self-report or chart review.

### Statistical analysis

Continuous variables are reported as median (interquartile range [IQR]) and categorical variables as percentages. Baseline characteristics were compared between groups using Kruskal-Wallis testing or linear model ANOVA for continuous variables and Pearson’s chi-squared tests for categorical variables, as appropriate. Rates of left ventricular (LV) dysfunction after myocardial infarction (MI), in-hospital mortality, and 30-day clinical events (mortality, all-cause readmission, heart failure [HF] readmission) were compared across glycemic groups with chi-squared testing. Post-MI LV dysfunction was defined as left ventricular ejection fraction of ≤40% during the index hospitalization, assessed by experienced cardiologists using biplane Simpson’s method on transthoracic echocardiography. Multivariable logistic regression adjusted for clinically relevant covariates selected a priori was performed to evaluate the association between newly detected diabetes, established diabetes, prediabetes, and euglycemia with clinical outcomes. Models were adjusted for age, sex, body mass index, tobacco use, history of hypertension, HF, prior MI, and prior stroke. Informed consent was obtained from all registry participants, and the study was approved by the Institutional Review Board at both sites of enrollment. Analyses were performed using SAS 9.4 (Cary, North Carolina, USA) and R Statistical Software (version 4.0.5, Vienna, Austria). Two-sided *P* values less than .05 were considered significant.

## Results

### Baseline characteristics

A total of 3,523 patients with STEMI were consecutively enrolled from January 2019 to February 2020. The median (IQR) age was 55 (46–62) years, with 40% of all participants aged 50 or younger; 16% were women, 55% had a BMI of ≥25 kg/m^2^, and more than 50% were current every day smokers at the time of presentation (Table 1).

**Table 1:** Selected Baseline Demographic and Clinical Characteristics of Included NORIN STEMI Participants, by Glycemic Status.


	EUGLYCEMIC (*N* = 1592)	PREDIABETES (*N* = 737)	NEWLY DETECTED DIABETES MELLITUS (*N* = 339)	ESTABLISHED DIABETES MELLITUS (*N* = 855)

**Demographic characteristics**				

Age, median (IQR), y	53 (45–60)	55 (45–62)	54 (45–60)	58 (50–64)

Women, *N* (%)	204 (13%)	103 (14%)	46 (14%)	215 (25%)

BMI,^a^ *N* (%)				

Underweight	49 (3%)	12 (2%)	12 (4%)	14 (2%)

Normal	743 (47%)	283 (38%)	130 (38%)	357 (42%)

Overweight	633 (40%)	363 (49%)	153 (45%)	369 (43%)

Obese	167 (11%)	79 (11%)	44 (13%)	115 (14%)

Hypertension	330 (21%)	132 (18%)	72 (21%)	488 (57%)

Atrial fibrillation	25 (2%)	13 (2%)	12 (4%)	26 (3%)

Heart failure	10 (1%)	7 (1%)	3 (1%)	16 (2%)

Hyperlipidemia	22 (1%)	6 (1%)	3 (1%)	46 (5%)

Prior stroke	16 (1%)	9 (1%)	4 (1%)	14 (2%)

Prior myocardial infarction	148 (9%)	107 (15%)	62 (18%)	120 (14%)

**Tobacco use, *N* (%)**				

Never	526 (33%)	259 (35%)	111 (33%)	419 (49%)

Former	127 (8%)	52 (7%)	21 (6%)	66 (8%)

Current—some days	60 (4%)	13 (2%)	8 (2%)	19 (2%)

Current—every day	879 (55%)	413 (56%)	199 (59%)	349 (41%)

**Education, *N* (%)**				

Illiterate	771 (48%)	409 (56%)	199 (59%)	413 (48%)

Middle School	470 (30%)	168 (23%)	60 (18%)	223 (26%)

High School	242 (15%)	116 (16%)	55 (16%)	133 (16%)

College Graduate	109 (7%)	44 (6%)	25 (7%)	86 (10%)

Aspirin	141 (9%)	82 (11%)	42 (12%)	117 (14%)

Statin	140 (9%)	81 (11%)	40 (12%)	118 (14%)


Abbreviations: BMI, body mass index. ^a^ Calculated as weight in kilograms divided by height in meters squared.

Among 855 (24%) patients with an established history of diabetes, 402 (47%) were treated with any antihyperglycemic therapy: 391 (46%) with metformin, 181 (21%) with sulfonylureas, 21 (2%) with GLP-1RA, 11 (1%) with insulin, 9 (1%) with thiazolidinediones, 6 (1%) with dipeptidyl-peptidase 4 inhibitors, and 1 (<1%) with SGLT2i. Baseline treatment with statin pharmacotherapy among patients with established diabetes was 14% (Supplementary Table 1).

### Impact of protocolized screening on dysglycemia prevalence among patients with STEMI

**Figure 1:**
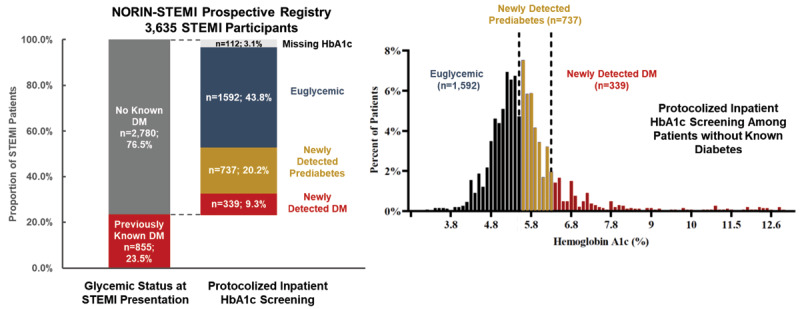
Results of Protocolized Inpatient HbA1c Screening among Patients Presenting with STEMI in North India, by Baseline Glycemic Status. Abbreviations: DM, diabetes mellitus; HbA1c, glycated hemoglobin; STEMI, ST-segment elevation myocardial infarction. The left panel represents the glycemic status of NORIN STEMI participants, before and after protocolized inpatient HbA1c screening. The panel to the right shows the distribution of HbA1c levels of NORIN STEMI participants without known dysglycemia at the time of presentation. Established diabetes was defined as a self-reported history of diabetes or clinical history by chart review. Euglycemia was defined as screening HbA1c <5.7%. Prediabetes and diabetes were defined as screening HbA1c 5.7–6.4% and ≥6.5%, respectively.

Median HbA1c of those with established diabetes (available in 826 of 855 [97%] participants) was 7.4% (IQR, 6.4–8.2%). Among patients without known diabetes, the median HbA1c (available in 2,668 participants) was 5.5% (IQR, 5.1–5.9%). Protocolized screening identified 737 (28%) to have prediabetes and 339 (13%) to have newly detected diabetes (Figure 1).

### Impact of dysglycemia on clinical outcomes

Rates of post-MI LV dysfunction were higher in those with prediabetes (49%), newly detected diabetes (53%), and established diabetes (48%) compared to euglycemic patients (42%) (*P* < 0.005 for each comparison). In-hospital mortality (5.6%, 5.1%, 10.3%, 4.3%) and 30-day mortality (8.1%, 7.6%, 14.4%, 6.6%) were similarly higher in patients in each dysglycemia group compared to euglycemic patients. However, only established diabetes was associated with a statistically significant increase in the risk of in-hospital mortality (*P* = 0.004) and 30-day mortality (*P* < 0.001) when compared with euglycemia. Thirty-day all-cause readmission (10.2%, 11.7%, 9.0%, 7.1%), and 30-day HF readmission (8.4%, 9.9%, 7.1%, 5.2%) were significantly higher among patients with prediabetes, newly detected diabetes, and established diabetes as compared with patients with euglycemia (*P* < 0.05 for each comparison) (Supplementary Table 2).

**Figure 2:**
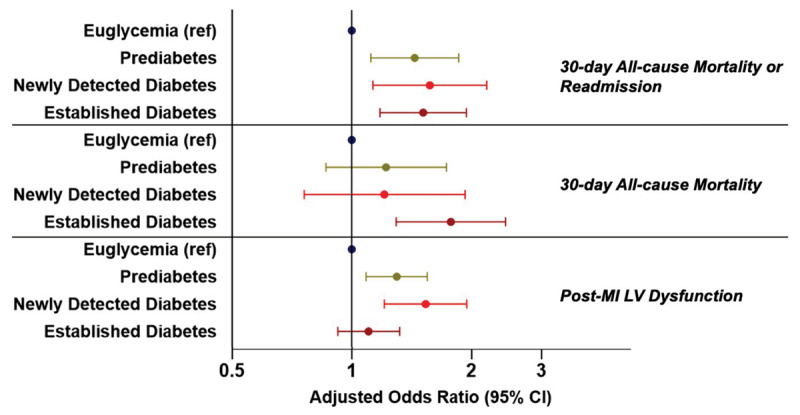
Clinical Outcomes of Patients Presenting with STEMI in North India, by Glycemic Status. Abbreviations: HF, heart failure; LV, left ventricle; MI, myocardial infarction. All models included adjustment for age, sex, body mass index, tobacco use, history of hypertension, heart failure, prior MI, and prior stroke.

After accounting for clinically relevant covariates, both prediabetes and newly detected diabetes, but not established diabetes, remained independently associated with higher odds of post-MI LV dysfunction (Figure 2). Prediabetes (adjusted OR 1.44; 95% CI 1.12–1.85), newly detected diabetes (adjusted OR 1.57; 95% CI 1.13–2.18), and established diabetes (adjusted OR 1.51; 95% CI 1.19–1.94) were independently associated with higher odds of composite 30-day mortality or readmission as compared with euglycemia. Patients with established diabetes, but not prediabetes or newly detected diabetes, experienced greater odds of 30-day mortality (adjusted OR 1.77; 95% CI 1.29–2.43).

## Discussion

In this prospective registry-based analysis of patients presenting with STEMI in North India, we found that protocolized HbA1c screening doubled the proportion of patients with known dysglycemia. Moreover, treatment with cardiometabolic risk reduction strategies, namely SGLT2i and GLP-1RA, was infrequent in patients with established diabetes. Consistent with prior studies [1,2,9], patients with dysglycemia experienced worse outcomes after STEMI, including higher all-cause mortality or readmission, as compared with patients with euglycemia. We additionally found that prediabetes and newly detected diabetes was associated with post-MI LV dysfunction. Overall, these data highlight gaps in dysglycemia surveillance and management among high-risk patients in North India. These missed opportunities are especially problematic in LMICs, where limited access to timely percutaneous coronary intervention [10] and secondary prevention medical therapy [11,12] may contribute to additional death and disability.

Coupled with population growth and aging, increasing rates of major cardiovascular risk factors have swung the burden of cardiovascular disease significantly toward LMICs, including India [13,14,15]. Although the contribution of dysglycemia to adverse cardiometabolic risk is well-established [1,16], prevention and treatment efforts in LMICs are challenged by high rates of underdiagnosis and limited access to important anti-hyperglycemic therapies [2,17]. The International Diabetes Federation has estimated that over 40 million people in India have unrecognized diabetes [2], and it has been estimated that approximately one in four households in India are unable to afford metformin [17]. More broadly throughout LMICs, fewer than 10% of persons with diabetes receive coverage of guideline-based comprehensive antihyperglycemic therapies [18]. The availability, affordability, and coverage of SGLT2i and GLP-1RA in LMICs is likely substantially more limited, which may explain the low rates of use observed in this study. Of note, SGLT2i have been included in the recently released 22nd WHO Essential Medicines List [19], representing an important step toward expanding use in areas of greatest need.

Importantly, newly diagnosed diabetes was independently associated with LV dysfunction in this NORIN STEMI analysis. As recently recognized in international heart failure (HF) clinical practice guidelines [20,21] and by the American Diabetes Association [22], diabetes is a robust but underappreciated contributor to the risk of LV dysfunction and incident HF even in the absence of other traditional cardiovascular risk factors. As in this registry, a common pathway to the development of LV dysfunction in patients with diabetes is via myocardial ischemia and infarction, driven in part by accelerated atherosclerosis owing to greater burden of atherogenic dyslipidemia, hyperinsulinemia-mediated inflammation, and endothelial dysfunction. Other key mechanisms are reviewed elsewhere [16,23]. Patients with diabetes who suffer MI are additionally at enhanced risk for adverse remodeling owing to a greater baseline burden of myocardial fibrosis, as well as persistent induction of maladaptive proinflammatory, neurohormonal, and oxidative pathways [16]. Despite wide underuse, GLP1-RA substantially reduce the risk of major adverse cardiovascular events (MACE) among patients with diabetes [24]. Early implementation of SGLT2i after acute MI holds substantial promise in averting subsequent progression to HF [25], potentially via abrogation of profibrotic, proinflammatory, and adverse neurohormonal pathways, among others [16,26,27]. This newly diagnosed diabetes cohort at time of MI is specifically under evaluation in the ongoing DAPA-MI (NCT04564742) and EMPACT-MI (NCT04509674) trials. Further, in observational analyses, use of GLP1-RA among survivors of MI with diabetes is associated with a nearly 30% lower risk of recurrent MACE compared to nonusers [28,29].

Consistent with another registry-based study [10], NORIN STEMI participants were young, with a median age of 55 years. This observation adds to the increasing recognition of acute MI in the young worldwide [7,9] and is likely attributable to an increasing cardiometabolic risk factor burden at early stages of life [2,30]. In addition to dysglycemia, more than half of NORIN STEMI participants had an elevated BMI, highlighting the rapid increase in overweight and obesity in LMICs over the past three decades [14,31]. Furthermore, rates of tobacco smoking were substantial, and baseline statin use at STEMI presentation was low irrespective of glycemic status. These findings are aligned with a recent international cross-sectional analysis of LMICs that revealed fewer than one in every ten statin-eligible individuals to be using a statin for the primary prevention of cardiovascular disease [12]. As statin use for secondary prevention has been observed to be only modestly higher – approximately 20% of all eligible individuals – in LMICs [12], further investigation will be necessary to determine whether the high rate (99–100%) of statin utilization observed among NORIN STEMI participants at discharge persists long-term. Given the increased disability-adjusted life years and lifetime medical costs associated with acute MI at a younger age, these data strongly stress the need for effective preventive strategies in LMICs.

Overall, achievement of sustainable development goals [32] to ameliorate the increasing burden of premature morbidity and mortality resulting from cardiovascular disease in LMICs will necessitate implementation of population-level systems that successfully prevent, identify, and treat dysglycemia and other cardiometabolic conditions. These data suggest that hospitalization for acute cardiovascular events may represent a high-yield opportunity to screen for, promote awareness around, and manage dysglycemia in LMICs.

### Limitations

Several limitations should be acknowledged. First, participant enrollment occurred at two large, urban, and government-funded academic hospitals in New Delhi, hence generalizability to other practice settings and LMICs may be limited. Second, only HbA1c testing was ascertained for glycemic assessment owing to its convenience and robustness as a marker of long-term dysglycemia, less affected by stress-induced hyperglycemia and insulin resistance owing to an acute cardiac event. Hence, we were unable to differentiate between type 1 and type 2 diabetes. It should be acknowledged that the diagnostic accuracy of HbA1c can be influenced by numerous factors [33,34], such as hemoglobinopathies, which were not explicitly accounted for in this analysis. However, HbA1c measurement has been shown to provide excellent sensitivity and specificity for diabetes diagnosis among high-risk Indian patients [35], and the assay employed in this registry has demonstrated clinical accuracy in the presence of common hemoglobin variants [36]. Repeat HbA1c levels were not available to affirm glycemic status. Third, baseline prediabetes status was unknown given limited documentation and awareness of this entity. Fourth, self-report and chart review were employed for assessment of baseline health and medications, which may be subject to recall bias, among other limitations of secondary data. Fifth, whether post-MI LVD was persistent at follow-up was not available, limiting ascertainment of the impact of LVD on clinical course. Finally, cause of death was not captured or separately adjudicated in NORIN STEMI.

## Conclusions

Among NORIN STEMI registry participants, protocolized HbA1c screening doubled the proportion of patients with known dysglycemia. Treatment with SGLT2i or GLP-1RA among patients with known diabetes was exceedingly rare, and patients with dysglycemia faced higher rates of post-MI LV dysfunction, readmission, and mortality out to 30 days. These data highlight important dysglycemia screening and management gaps among patients presenting with STEMI in North India.

## Additional Files

The additional files for this article can be found as follows:

10.5334/gh.1140.s1 Supplementary Table 1. Full Baseline Characteristics of Included NORIN STEMI Participants, by Glycemic Status.

10.5334/gh.1140.s2 Supplementary Table 2. Outcomes by Glycemic Status in NORIN STEMI.
