# Supplementary material for: Missed Opportunities for Screening and Management of Dysglycemia among Patients Presenting with Acute Myocardial Infarction in North India: The Prospective NORIN STEMI Registry
Source: Glob Heart. 2022 Aug 12;17(1):54. doi: 10.5334/gh.1140 (PMC9374010; doi:10.5334/gh.1140)
Supplement: Supplementary Table 1. — Full Baseline Characteristics of Included NORIN STEMI Participants, by Glycemic Status. [file gh-17-1-1140-s1.pdf]

Supplemental Material

Supplementary Table 1: Full Baseline Characteristics of Included NORIN STEMI Participants, by Glycemic Status

	**Euglycemic** (N=1592)	**Prediabetes** (N=737)	**Newly Detected Diabetes Mellitus** (N=339)	**Established Diabetes Mellitus** (N=855)
**Age, median (IQR), y**	53 (45-60)	55 (45-62)	54 (45-60)	58 (50-64)
≥ 60 years, N (%)	180 (11%)	68 (9%)	39 (12%)	21 (3%)
50-59 years, N (%)	424 (27%)	179 (24%)	73 (22%)	170 (20%)
40-49 years, N (%)	469 (30%)	225 (31%)	110 (32%)	267 (31%)
<40 years, N (%)	519 (33%)	265 (36%)	117 (35%)	397 (46%)
Women, N (%)	204 (13%)	103 (14%)	46 (14%)	215 (25%)
Direct	88 (6%)	51 (7%)	20 (6%)	66 (8%)
Referral	1504 (94%)	686 (93%)	319 (94%)	789 (92%)
Ambulance	73 (5%)	26 (4%)	9 (3%)	67 (8%)
Public-Transport	533 (34%)	335 (45%)	131 (39%)	305 (36%)
Self/Family	986 (62%)	376 (51%)	199 (59%)	483 (57%)
Non-PCI Center (Clinic)	291 (18%)	111 (15%)	36 (11%)	128 (15%)
Non-PCI Center (Hospital)	1203 (76%)	580 (79%)	288 (85%)	644 (75%)
PCI Center	98 (6%)	46 (6%)	15 (4%)	83 (10%)
Diabetes Mellitus	0 (0%)	0 (0%)	0 (0%)	855 (100%)
Hypertension	330 (21%)	132 (18%)	72 (21%)	488 (57%)
Cancer	1 (0%)	1 (0%)	0 (0%)	5 (1%)
Atrial Fibrillation	25 (2%)	13 (2%)	12 (4%)	26 (3%)
Hemodialysis	1 (0%)	0 (0%)	0 (0%)	0 (0%)
Heart Failure	10 (1%)	7 (1%)	3 (1%)	16 (2%)
Hyperlipidemia	22 (1%)	6 (1%)	3 (1%)	46 (5%)

Prior cerebrovascular accident	16 (1%)	9 (1%)	4 (1%)	14 (2%)
Prior myocardial infarction	148 (9%)	107 (15%)	62 (18%)	120 (14%)
Prior coronary artery bypass grafting	3 (0%)	1 (0%)	0 (0%)	2 (0%)
Peripheral artery disease	0 (0%)	0 (0%)	0 (0%)	4 (1%)
**Tobacco use**				
Never	526 (33%)	259 (35%)	111 (33%)	419 (49%)
Former	127 (8%)	52 (7%)	21 (6%)	66 (8%)
Current – some days	60 (4%)	13 (2%)	8 (2%)	19 (2%)
Current – every day	879 (55%)	413 (56%)	199 (59%)	349 (41%)
**Education**				
Illiterate	771 (48%)	409 (56%)	199 (59%)	413 (48%)
Middle School	470 (30%)	168 (23%)	60 (18%)	223 (26%)
High School	242 (15%)	116 (16%)	55 (16%)	133 (16%)
College Graduate	109 (7%)	44 (6%)	25 (7%)	86 (10%)
**Occupation**				
At home	191 (12%)	89 (12%)	39 (12%)	200 (23%)
Manual Laborer	566 (36%)	291 (40%)	133 (39%)	187 (22%)
Professional	175 (11%)	82 (11%)	66 (20%)	88 (10%)
Retired	75 (5%)	40 (5%)	17 (5%)	81 (10%)
Self-Employed	443 (28%)	177 (24%)	50 (15%)	216 (25%)
Student	3 (0%)	1 (0%)	2 (1%)	0 (0%)
Unemployed	139 (9%)	57 (8%)	32 (9%)	83 (10%)
**Alcohol**				
Current - Every Day	142 (9%)	73 (10%)	42 (12%)	59 (7%)
Current - Some Days	218 (14%)	69 (9%)	37 (11%)	63 (7%)
Former	78 (5%)	24 (3%)	20 (6%)	54 (6%)
Never	1154 (73%)	571 (78%)	240 (71%)	679 (79%)

**Baseline Medications**				
Aspirin	141 (9%)	82 (11%)	42 (12%)	117 (14%)
Statin	140 (9%)	81 (11%)	40 (12%)	118 (14%)
Beta Blocker	174 (11%)	85 (12%)	42 (12%)	162 (19%)
Calcium Channel Blocker	70 (4%)	25 (3%)	21 (6%)	75 (9%)
Systolic blood pressure < 90 mmHg	28 (2%)	22 (3%)	13 (4%)	20 (2%)
Heart rate > 100 beats/min	153 (10%)	67 (9%)	31 (9%)	110 (13%)
**BMI**[a]				
Underweight	49 (3%)	12 (2%)	12 (4%)	14 (2%)
Normal	743 (47%)	283 (38%)	130 (38%)	357 (42%)
Overweight	633 (40%)	363 (49%)	153 (45%)	369 (43%)
Obese	167 (11%)	79 (11%)	44 (13%)	115 (14%)
Serum creatinine (missing=4)	1.0 (0.8-1.2)	1.0 (0.8-1.2)	1.0 (0.8-1.2)	1 (0.9-1.2)
Hemoglobin (missing=1)	12.9 (11.8-14.0)	12.8 (11.8-13.7)	12.8 (11.9-13.8)	12.7 (11.7-13.6)
Total cholesterol (missing=582)	154 (126-183)	149 (122-180)	153 (124-180)	150 (121-187)
Triglycerides (missing=581)	127 (95-167)	128 (93-168)	127 (88-172)	136 (100-181)
Low-density lipoprotein cholesterol (missing=581)	81 (62-105)	84 (62-109)	86 (66-111)	73 (56-96)
High-density lipoprotein cholesterol (missing=586)	37 (31-45)	37 (30-44)	37 (32-44)	37 (30-44)
Hemoglobin A1C (%) (missing=29)	5.2 (4.9-5.4)	5.9 (5.8-6.1)	7.2 (6.8-9.0)	7.4 (6.4-8.2)
Anterior Wall MI	856 (54%)	394 (54%)	186 (55%)	471 (55%)
Inferior Wall MI	685 (43%)	318 (43%)	145 (43%)	365 (43%)
Lateral Wall MI	21 (1%)	5 (1%)	0 (0%)	10 (1%)
Posterior Wall MI	9 (1%)	4 (1%)	1 (0%)	2 (0%)
Anterior Wall, Inferior Wall	6 (0%)	5 (1%)	3 (1%)	3 (0%)

Anterior Wall, Lateral Wall	10 (1%)	6 (1%)	0 (0%)	3 (0%)
Inferior Wall, Lateral Wall	2 (0%)	1 (0%)	4 (1%)	0 (0%)
Inferior Wall, Posterior Wall	3 (0.2%)	4 (0.5%)	0 (0.0%)	1 (0.1%)
Ventricular fibrillation	8 (0%)	3 (0%)	1 (0%)	12 (1%)
Regional Wall Motion Abnormality	1217 (76%)	631 (86%)	286 (84%)	672 (79%)
**Ejection Fraction**				
≥50%	231 (15%)	79 (11%)	43 (13%)	110 (13%)
41-49%	696 (44%)	298 (40%)	115 (34%)	336 (39%)
31-40%	561 (35%)	300 (41%)	148 (44%)	320 (37%)
≤ 30%	104 (7%)	60 (8%)	33 (10%)	89 (10%)
**Mitral Regurgitation**				
None	1139 (72%)	515 (70%)	235 (69%)	575 (67%)
Mild	378 (24%)	178 (24%)	85 (25%)	234 (27%)
Moderate	71 (5%)	41 (6%)	18 (5%)	43 (5%)
Severe	4 (0%)	3 (0%)	1 (0%)	3 (0%)
Left Ventricle Thrombus	3 (0%)	1 (0%)	1 (0%)	0 (0%)
Ventricular Septal Defect	13 (1%)	12 (2%)	3 (1%)	7 (1%)
Left Ventricular Aneurysm	1 (0%)	0 (0%)	0 (0%)	1 (0%)
Acute Mitral Regurgitation	3 (0%)	3 (0%)	2 (1%)	2 (0%)
**Medications on Hospital Arrival**				
Aspirin	1585 (100%)	736 (100%)	338 (100%)	850 (99%)
Statin	1585 (100%)	735 (100%)	338 (100%)	849 (99%)
P2Y12 Inhibitors	1583 (99%)	737 (100%)	338 (100%)	850 (99%)
Clopidogrel	1466 (92%)	706 (96%)	323 (95%)	800 (94%)
Ticagrelor	83 (5%)	10 (1%)	0 (0%)	33 (4%)
Prasugrel	34 (2%)	21 (3%)	15 (4%)	17 (2%)

None	9 (1%)	0 (0%)	1 (0%)	5 (1%)
Beta Blocker	383 (24%)	75 (10%)	21 (6%)	163 (19%)
Anticoagulation				
Unfractionated heparin	1072 (67%)	471 (64%)	204 (60%)	541 (63%)
Low-molecular weight heparin	10 (1%)	1 (0%)	0 (0%)	1 (0%)
None	510 (32%)	265 (36%)	135 (40%)	313 (37%)
Angiography	1188 (74%)	513 (70%)	221 (65%)	593 (69%)
Arterial Access Site				
Femoral	1138 (96%)	502 (98%)	216 (98%)	565 (95%)
Radial	39 (4%)	5 (2%)	2 (2%)	16 (5%)
Culprit Artery				
LAD	430 (36%)	247 (48%)	125 (57%)	260 (44%)
LCX	100 (8%)	39 (8%)	17 (8%)	49 (8%)
LMCA	5 (0%)	0 (0%)	0 (0%)	2 (0%)
Normal	1 (0%)	0 (0%)	0 (0%)	0 (0%)
RCA	267 (22%)	176 (34%)	72 (33%)	143 (24%)
SCAD	0 (0%)	1 (0%)	1 (0%)	1 (0%)
Number of Vessels				
0	13 (1%)	4 (1%)	4 (1%)	7 (1%)
1	799 (67%)	361 (70%)	175 (79%)	375 (63%)
2	286 (24%)	110 (21%)	31 (14%)	146 (25%)
3	91 (8%)	38 (7%)	11 (5%)	66 (11%)
Percutaneous Coronary Intervention	1080 (68%)	473 (64%)	204 (60%)	542 (63%)
Hours to PCI, Median (IQR)	4 (2-4)	4 (2-4)	4 (2-4)	4 (2-4)
**Medications on Discharge**				
Aspirin	1507 (100%)	680 (100%)	315 (100%)	752 (100%)

Statin	1499 (99 %)	679 (100%)	314 (100%)	748 (100%)
P2Y12 Inhibitor	1499 (99%)	679 (100%)	314 (100%)	747 (99%)
Beta Blocker	1480 (98%)	672 (99%)	313 (99%)	734 (98%)
Calcium Channel Blocker	44 (3%)	17 (3%)	4 (1%)	22 (3%)
ACEi/ARB	1352 (90%)	587 (86%)	267 (85%)	657 (87%)

Abbreviations: ACEi, angiotensin-converting enzyme inhibitor; ARB, angiotensin receptor blocker; BMI, body mass index; HF, heart failure; IQR = interquartile range; MI, myocardial infarction; PCI, percutaneous coronary intervention; LAD, left anterior descending artery; LCX, left circumflex artery; LMCA, left main coronary artery; RCA, right coronary artery; SCAD, spontaneous coronary artery dissection

[a] Calculated as weight in kilograms divided by height in meters squared.
