# Supplementary material for: Missed Opportunities for Screening and Management of Dysglycemia among Patients Presenting with Acute Myocardial Infarction in North India: The Prospective NORIN STEMI Registry
Source: Glob Heart. 2022 Aug 12;17(1):54. doi: 10.5334/gh.1140 (PMC9374010; doi:10.5334/gh.1140)
Supplement: Supplementary Table 2. — Outcomes by Glycemic Status in NORIN STEMI. [file gh-17-1-1140-s2.pdf]

Supplementary Table 2: Outcomes by Glycemic Status in NORIN STEMI

	**Euglycemic** (N=1592)	**Prediabetes** (N=737)	*P* value[a]	**Newly Detected Diabetes Mellitus** (N=339)	*P* value[a]	**Established Diabetes Mellitus** (N=855)	*P* value[a]
Post-MI LV Ejection Fraction ≤40%	665 (42%)	360 (49%)	<0.001	181 (53%)	<0.001	409 (48%)	0.004
In-hospital Mortality	68 (4%)	40 (6%)	0.20	17 (5%)	0.56	86 (10%)	<0.001
30-day Mortality	99 (7%)	56 (8%)	0.21	25 (8%)	0.47	107 (13%)	<0.001
30-day All-cause Readmission	88 (7%)	62 (10%)	0.03	34 (12%)	0.02	56 (9%)	<0.001
30-day HF Readmission	72 (5%)	53 (8%)	0.007	30 (10%)	0.003	49 (7%)	<0.001

Abbreviations: HF, heart failure; LV, left ventricle; MI, myocardial infarction

[a] Pearson's Chi-squared test compared to euglycemic patients. Reported *P*-values are two-sided.
